# The Effects of Exercise Training on Functional Capacity and Quality of Life in Patients with Rheumatoid Arthritis: A Systematic Review

**DOI:** 10.3390/jcdd11060161

**Published:** 2024-05-22

**Authors:** Amalia Athanasiou, Ourania Papazachou, Nikoletta Rovina, Serafim Nanas, Stavros Dimopoulos, Christos Kourek

**Affiliations:** 1 Clinical Ergospirometry, Exercise & Rehabilitation Laboratory, 1st Critical Care Medicine Department, Evangelismos Hospital, National and Kapodistrian University of Athens, 10676 Athens, Greece; ama.athanasiou@gmail.com (A.A.); ranpapaza@yahoo.gr (O.P.); sernanas@gmail.com (S.N.); stdimop@gmail.com (S.D.); 2 Department of Cardiology, “Helena Venizelou” Hospital, 10676 Athens, Greece; 3 1st Department of Respiratory Medicine, Sotiria Chest Hospital, School of Medicine, National and Kapodistrian University of Athens, 11527 Athens, Greece; nikrovina@med.uoa.gr; 4 Cardiac Surgery Intensive Care Unit, Onassis Cardiac Surgery Center, 17674 Athens, Greece; 5 Department of Cardiology, 417 Army Share Fund Hospital of Athens (NIMTS), 11521 Athens, Greece

**Keywords:** rheumatoid arthritis, peak VO_2_, quality of life, aerobic exercise, anaerobic exercise

## Abstract

Rheumatoid arthritis (RA) is an autoimmune disease characterized by chronic inflammation. The purpose of this systematic review is to evaluate the effectiveness of exercise training on functional capacity and quality of life (QoL) in patients with RA. We performed a search in four databases, selecting clinical trials that included community or outpatient exercise training programs in patients with RA. The primary outcome was functional capacity assessed by peak VO_2_ or the 6 min walking test, and the secondary outcome was QoL assessed by questionnaires. Seven studies were finally included, identifying a total number of 448 patients. The results of the present systematic review show a statistically significant increase in peak VO_2_ after exercise training in four out of seven studies. In fact, the improvement was significantly higher in two out of these four studies compared to the controls. Six out of seven studies provided data on the patients’ QoL, with five of them managing to show statistically significant improvement after exercise training, especially in pain, fatigue, vitality, and symptoms of anxiety and depression. This systematic review demonstrates the beneficial effects of exercise training on functional capacity and QoL in patients with RA.

## 1. Introduction

Rheumatoid arthritis (RA) is a chronic autoimmune inflammatory disorder characterized by inflammatory arthritis and extra-articular involvement [1]. In particular, some symptoms of rheumatoid arthritis are the presence of pain, tenderness, and redness and swelling in certain joints, usually in the fingers, wrists, or metatarsals, while there is often symmetry in the joint involvement [2]. It is a fairly common disease worldwide and it is estimated to affect approximately 0.24% to 1% of the population. In Europe and North America, its prevalence is 0.5 to 1.0% [3]. In addition, it affects at least twice as many women as men, and although the onset of the disease can occur at any age, the greatest risk of its occurrence is at the age of 50 [4]. Patients with RA frequently present exercise intolerance [5], increased cardiovascular risk [5], and decreased quality of life (QoL) [6].

Exercise has a significant role in preventing or improving vascular endothelial function [7]. Endothelial nitric oxide synthase (eNOS) plays an essential role in the regulation of endothelial function and acts as a principal regulator of vascular tone and homeostasis [8,9]. Exercise affects vascular reactivity in the coronary and skeletal muscle circulations through upregulation of nitric oxide (NO) release and endothelium-dependent hyperpolarization-mediated responses resulting from increases in blood flow and thus shear stress [10]. In recent years, evidence has accumulated confirming that NO release by endothelial cells can be chronically increased (e.g., by estrogen, exercise) and decreased (e.g., by oxidative stress) by aging and during vascular diseases (e.g., diabetes and hypertension) [11]. It has been shown that aerobic exercise, as well as its combination with resistance exercise, improves maximal oxygen uptake (peak VO_2_), vascular endothelial function, or QoL in patients with cardiovascular comorbidities [12,13,14,15] and autoimmune rheumatic diseases [16]. Physical activity or moderate intensity exercise have profound anti-inflammatory effects in patients with chronic diseases or RA [17,18]. As far as the possible underlying molecular mechanisms are concerned, recent studies have investigated the possible role in heart failure (HF) of sirtuins, a family of nicotinamide adenine dinucleotide (NAD+)-dependent deacetylases, among which sirtuin 1 (Sirt1) is the best characterized member [19,20]. Sirtuin 1 is involved in biological processes strongly related to HF, including oxidative stress and cellular senescence. It also plays a crucial role in angiotensin II-induced vascular remodeling and inflammatory response by modulating the expression of certain cytokines [19,20]. It is not surprising, therefore, that exercise is recognized as an activator of Sirt1. High frequency of training induces systemic vascular adaptations and increases the effect of aerobic exercise on endothelial function compared to a low training frequency [13]. In addition, high-intensity interval training increases endothelial function to a greater extent than moderate-intensity training [13]. Nevertheless, the impact of exercise training on functional capacity in patients with RA has not been extensively studied in the literature.

The aim of the present systematic review is to evaluate and compare the effect of combined exercise training programs on functional capacity and QoL in patients with RA.

## 2. Materials and Methods

### 2.1. Search Strategy

A search for clinical trials or randomized controlled trials (RCTs) was performed in Pubmed, Cochrane, PEDro, and Embase databases between 28 June 2023 and 10 July 2023. Articles that resulted from the search were selected based on specific criteria: English language and clinical studies or RCTs. Specific terms including (“Exercise” OR “Exercise Training” OR “Rehabilitation” OR “Breathing Exercise”) AND (“Rheumatoid Arthritis” OR “Arthritis” OR “Rheumatic Disease” OR “Rheumatics”) were used for the search. This review was performed in accordance with the PRISMA (Preferred Reporting Items for Systematic Reviews and Meta-Analyses) guidelines. Duplicates were removed from the initial number of studies, and the rest were evaluated twice. The search and evaluation of studies were performed by the principal investigator and verified and assessed for validity and accuracy by an independent investigator. Final evaluation and approval were performed by a third independent researcher. The data used for each study included demographic characteristics of the intervention and control group, details on training protocols, key endpoints, and results.

### 2.2. Study Selection Criteria

The inclusion criteria were as follows: (1) clinical trials or randomized clinical trials that included an interventional and a control group; (2) patients with RA, under stable treatment and without hospitalizations in the last three months; (3) aged ≥18 years; (4) aerobic or resistance exercise training or combined exercise protocols; (5) duration of exercise training ≥2 weeks; and (6) studies that included results of exercise training on peak VO_2_ or the 6 min walking test and/or QoL.

The exclusion criteria were as follows: (1) systematic reviews, guidelines, abstracts, and meta-analyses; (2) patients with comorbidities of increased severity and symptoms, such as moderate to severe HF (New York Heart Association (NYHA) class III–IV), moderate to severe chronic obstructive pulmonary disease (COPD), asthma, and vasculitis; (3) studies using a different intervention than exercise; and (4) studies investigating the acute effect of exercise.

### 2.3. Quality Assessment

A quality assessment was performed using the Physiotherapy Evidence Database (PEDro Scale). This scale consists of a checklist of 11 yes/no questions related to internal validity and statistical information provided. Each study receives 1 unit for each criterion it meets. The maximum score is 10/10 (criterion 1 regarding eligibility criteria is not included in the final score, as it refers to external validity). High-quality studies are those with a score of 6–10/10, moderate 4–5/10, and low ≤3/10.

### 2.4. Outcome Measures

The primary outcome was functional capacity assessed by peak VO_2_ or the 6 min walking test, while the secondary outcome was the assessment of QoL through questionnaires (e.g., Short-Form Health Survey 36 (SF-36)). Both outcomes were evaluated at baseline and post-intervention.

## 3. Results

### 3.1. Search Results

**Figure 1:**
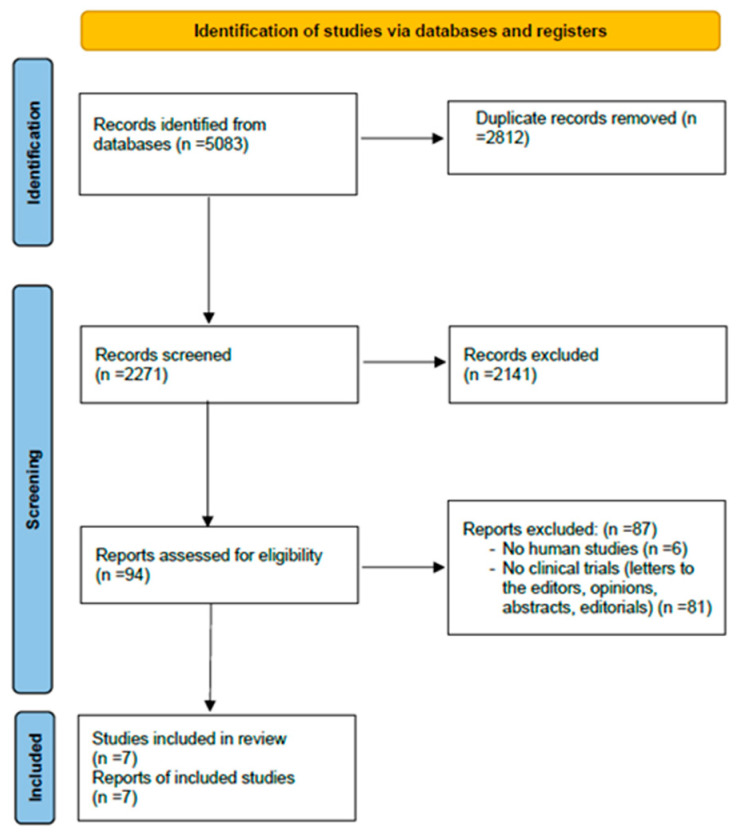
PRISMA flowchart including the screening results of the systematic review.

The results of the research and screening are illustrated in the PRISMA flowchart. (Figure 1). The initial search strategy identified 5083 articles from PubMed, Cochrane, PEDro, and Embase databases. The removal of duplicate publications excluded 2812 articles. Subsequently, from the screening of 2271 studies, 2141 were excluded after title or abstract assessment. Ninety-four studies were assessed for eligibility, of which 87 were excluded due to exclusion criteria. Therefore, seven studies met the inclusion criteria and were finally included in the review [21,22,23,24,25,26,27].

### 3.2. Assessment of the Methodological Quality of the Studies

The methodological quality of the studies (RCTs and clinical trials) was assessed using the PEDro scale. The scores on the PEDro scale ranged from 4 to 7 (Table 1). None of the studies had an overall score of less than 4. One of the seven studies had an overall score of 4 and was classified as good. The remaining seven studies had a total score of 5 to 7 and are characterized as very good. The lowest scoring area in all the studies was participant and therapist blindness.

### 3.3. Characteristics of Participants

The total number of participants was 448, with the majority of them being females (145 vs. 50 males). Aerobic exercise was performed by 232 patients, 37 patients performed a combination of aerobic exercise and resistance exercise, and the remaining 179 belonged to the control group. The mean age of the participants ranged from 44 to 55 years, while the mean time since the diagnosis of RA ranged from 2 to 35 years. The mean peak VO_2_ (mL/kg/min) ranged from 21.10 to 34.2. The disease activity score was low to moderate, as the mean value ranged from 2.9 to 4.1, while the body mass index (BMI) was high, with a range of 26.1 kg/m^2^ to 28.8 kg/m^2^. The demographics and baseline characteristics are outlined in Table 2.

### 3.4. Exercise Training Protocols 

The seven studies used specific exercise protocols. Six studies consisted of one intervention group and one control group, while one consisted of two intervention groups and one control group. Aerobic exercise was used as an intervention in all the studies, while combined aerobic and resistance exercise was performed in five of them. In one study, aerobic exercise was performed through the form of dancing, and in another study, it was performed in water. The duration of the intervention ranged from 3 to 6 months (3 months in five studies, 2 months in one study, and 6 months in one study), while the frequency of exercise training was from 2 to 3 times a week. Each training session had a duration between 35 and 90 min. The controls received usual care that varied among the studies. Specifically, in Bilberg et al.’s [23] and Stavropoulos-Kalinoglou et al.’s studies [26], the patients continued their daily activities, which included the home exercise program introduced to them on admission to the clinic. In Neuberger et al.’s [24] study, the patients were asked to remain at the same level of fitness as they had at the baseline measurement, while in Azeez et al.’s study [27], the standard care involved advice on the benefits of exercise in rheumatoid arthritis and outlining recommendations for physical activity in older adults. Finally, in some studies [21,22,25], the usual care was not further explained. 

The cardiopulmonary exercise testing (CPET) characteristics are highlighted in Table 2 for each study.

### 3.5. Effect of Exercise Training on Cardiorespiratory Fitness

The peak VO_2_ improved significantly within the intervention group after exercise training in four out of seven studies [21,25,26,27] (Table 3). In fact, the improvement was significantly higher in two out of these four studies compared to the controls [25,26]. In Noreu et al.’s study [21], the peak VO_2_ was found to increase in the intervention group by 13% (22.2 ± 7.4 to 25.0 ± 7.2 mL/kg/min, *p* ≤ 0.01), while in the control group, it remained unchanged (22.7 ± 6.5 to 23.8 ± 5.7 mL/kg/min, *p* > 0.05). In the study of Bilberg et al. [23], there was no improvement in the peak VO_2_ in either the intervention or the control group after exercise training. Similar results were presented in the research performed by Neuberger et al. [24], where, although there was significant improvement within each exercise group and not within the control group, no statistically significant difference between the intervention groups and the control group was observed (*p* > 0.05). In the study of Breedland et al. [25], there was a significant improvement in the intervention group (31.52 ± 10.17 to 35.34 ± 11.33 mL/kg/min, *p* ≤ 002), while no improvement was noticed in the control group (25.99 ± 6.07 to 25.55 ± 6.36 mL/kg/min), with a statistically significant difference between the two groups (intervention group: +12.1% versus control group: −1.7%; *p* < 0.05). In the study of Stavropoulos-Kalinoglou et al. [26], the intervention group showed a significant improvement (*p* < 0.002), with no improvement in the control group. Moreover, a statistically significant difference was observed between the groups (*p* = 0.002). In the research of Rintala et al. [22], both groups improved their peak VO_2_ (intervention group from 27.9 ± 7.1 to 29.5 ± 7.0 mL/kg/min and control group from 25.7 ± 5.1 to 27.1 ± 5.8 mL/kg/min; *p* < 0.05); however, there was no statistically significant difference between them (*p* = 0.204). Finally, in the study of Azeez et al. [27], the intervention group showed a statistically significant improvement [23.2 (16–88) to 27.6 (14–75) mL/kg/min, *p* = 0.002), while the control group showed no difference [26.1 (14–83) to 27.6 (18–65) mL/kg/min, *p* = 0.313). However, no statistically significant difference was observed between the two groups.

### 3.6. Effect of Exercise Training on Quality of Life

Six out of seven studies provided data on the patients’ QoL before and after exercise training [21,23,24,25,26,27] (Table 3). Five of them managed to show statistically significant improvement in QoL after exercise training [21,23,24,26,27]. Specifically, Noreu et al. [21] presented a statistically significant improvement in pain (4.37 ± 2.15 to 3.47 ± 1.85), mobility (0.46 ± 1.26 169 to 1.25 ± 2.08), household activities (1.01 ± 1.03 to 0.77 ± 0.89), depression (2.05 ± 1.56 to 1.18 ± 1.29), and anxiety (4.22 ± 1.50 to 3.07 ± 1.74) on the Abnormal Involuntary Movement Scale (AIMS) questionnaire, and tension (7.32 ± 6.35 to 4.11 ± 4.41), vigor (16.47 ± 4.26 to 20.37 ± 4.28), depression (8.74 ± 7.65 to 4.74 ± 5.96), fatigue (7.89 ± 5.86 to 5.68 ± 4.77), and total score (22.4 ± 27.7 to 2.6 ± 23.1) on the POMS questionnaire within the intervention group (*p* < 0.05), while in the control group, all these parameters remained unchanged, except for the depression score (11.50 ± 12.64 to 6.70 ± 6.62) of the Profile of Mood States (POMS) questionnaire. In the clinical trial of Bilberg et al. [23], the intervention group showed a statistically significant improvement in functionality (56.0 ± 20.9 to 64.7 ± 20.0, *p* = 0.001), physical pain (40.7 ± 21.0 to 50.8 ± 23.4, *p* < 0.05), vitality (41.5 ± 23.9 to 51.8 ± 22.6, *p* = 0.01), physical condition (33.0 ± 9.6 to 37.1 ± 10.5, *p* = 0.01), and mental health (68.4 ± 23.5 to 77.5 ± 17.6, *p* = 0.01) after a follow-up at 6 months, according to the SF-36 questionnaire. However, only vitality improved significantly in the intervention group compared to the controls (*p* = 0.021). In addition, physical health (2.6 ± 1.5 to 2.1 ± 1.4, *p* = 0.01) and the Health Assessment Questionnaire (HAQ) score (0.9 ± 0.5 to 0.7 ± 0.5, *p* = 0.05) of the Arthritis Impact Measurement Scales 2 (AIMS2) questionnaire improved statistically significantly only in the intervention group. This improvement in the HAQ score was statistically higher in the intervention group compared to the control group (*p* = 0.045). In the research by Neuberger et al. [24], according to the POMS, McGill Pain, and the Center for Epidemiologic Studies Depression Scale (CES-D) questionnaires, there was a reduction in symptoms of fatigue, pain, and depression within the intervention groups, as well as between the intervention groups and controls (*p* < 0.04). Overall symptoms such as fatigue (1.53 ± 0.98 to 1.35 ± 1.08), pain (4.67 ± 2.14 to 4.05 ± 2.24), and depression (0.59 ± 0.67 to 0.49 ± 0.62) decreased significantly after exercise training. In the research by Stavropoulos-Kalinoglou et al. [26], there was a statistically significant difference in the Disease Activity Score (DAS28) (3.5 ± 1.2 to 2.7 ± 0.7, *p* < 0.05) and HAQ (1.4 ± 0.8 to 0.9 ± 0.6, *p* < 0.001) within the intervention group, while the control group did not show improvement in the parameters above. The DAS28 and HAQ score improvement was significantly higher in the intervention group than the controls (*p* = 0.008 and *p* = 0.003, respectively). Finally, Azeez et al. [27] showed improvements in the HAQ (0.5 (0.0–2.4) to 0.25 (0.0–2.5), *p* = 0.05) and the Global Fatigue Index (GFI) score (13.2 (6.4–34.1) to 10.9 (6.5–37.5), *p* = 0.047) within the intervention group, but only the GFI score was significantly better compared to the controls.

On the contrary, Breedland et al. [25] failed to show significant differences between the intervention and control groups according to the Dutch version of the Arthritis Impact Measurement Scales-2 (Dutch-AIMS2) and the Arthritis Self-Efficacy Scale (ASES) questionnaires. 

## 4. Discussion

In the present systematic review, we showed that various modalities of aerobic exercise, as well as its combination with resistance training, resulted in the improvement of exercise capacity, as reflected through peak VO_2_ and QoL, as assessed via questionnaires (SF-36, AIMS, GFI, HAQ) in patients with RA. The new insight of our systematic review is the assessment of exercise training effects on functional capacity, specifically on peak VO_2_. Peak VO_2_ is considered as the principal outcome variable of cardiopulmonary exercise testing, which is the gold standard method for assessing cardiovascular functional capacity [28,29,30].

The vascular endothelium plays an important role in the cardiovascular system in maintaining blood circulation, regulating vascular tone, and promoting microvascular permeability, angiogenesis, and inflammatory response [3]. Endothelial dysfunction is an early onset in the pathogenesis of cardiovascular diseases [31]. Patients with RA usually present low maximal aerobic capacity and, therefore, low endurance, rapid fatigue, and reduced QoL [3,32,33]. On the other hand, exercise training induces a repeated increase in shear stress, which leads in an increase of the bioavailability of nitric oxide (NO) and a favorable effect on the oxidative balance [34]. Nitric oxide regulates central biological processes in almost all tissues, cells, and organs of the body [35]. Additionally, it can induce systemic molecular pathways, linked to angiogenesis and chronic anti-inflammatory action with subsequent improvement of endothelial function [35]. All the above beneficial effects of exercise at the microcellular level correspond with improvement of functional capacity and QoL in patients with cardiovascular disease or other comorbidities [36,37,38,39,40]. Therefore, systematic exercise could induce beneficial effects in patients with RA, including the increase of peak VO_2_, as well as the improvement of the vascular endothelial function, physical condition, body strength, mental health, and, finally, their QoL [41]. 

This systematic review consists of five RCTs and two clinical trials. Four of these studies found a statistically significant increase in peak VO_2_ within the intervention group, but not in the controls [21,25,26,27], and this increase was significantly higher in the intervention group compared to the controls in two of these four studies [25,26]. The remaining studies did not manage to show a statistically significant difference between the groups [21,22,23,24,27]. One of the main reasons that improvement was not achieved in these studies was the small number of participants, which may have lowered the power of the analyses. Moreover, a higher baseline peak VO_2_ of patients with RA before exercise training, lack of randomization in clinical trials in peak VO_2_ between the two groups, and/or a lower intensity of exercise protocols may be other reasons that led to non-significant differences in functional capacity between the groups. Instead, the combination of aerobic and resistance training with higher intensity resulted in statistically significant improvement in peak VO_2_.

An equally significant aspect of rehabilitation in patients with RA is the QoL, which is directly affected by the symptoms of the disease. In order to improve the QoL in patients with RA, it is necessary to increase their functional capacity and reduce their symptoms, fatigue, and pain. Regular physical exercise seems to contribute significantly to this aim, as it particularly modifies the metabolic potential, the morphology, and the physiology of the skeletal muscles, thus producing a number of beneficial effects on exercise tolerance and the QoL of patients with RA [21,23,24,26,27]. In our systematic review, there was improvement in the QoL in most studies [21,23,24,26,27]. Many questionnaires have been widely used in the assessment of the QoL in various clinical syndromes. In RA, investigators used the AIMS, AIMS2, Dutch AIMS2, ASES, SF-36, POMS, MAF, HAQ, GFI, McGill, and CES-D tools, as well as the activity of the disease (DAS28) in order to assess QoL. All these tools have proven to be reliable in different studies and are also being used to assess other clinical syndromes except for RA.

Acute exercise is another significant aspect that has been shown to offer several beneficial effects for individuals with RA. Specifically, Pereira Nunes Pinto AC et al. [42] showed that a 25 min single resistance exercise session including knee extension, knee flexion, hip abduction, and hip adduction, with one set of 12 repetitions at 50% of one repetition maximum (1 RM) and one set of eight repetitions at 75% of 1 RM decreased interleukin 1-beta (IL-1β) and increased anti-inflammatory cytokines IL-1 receptor antagonist (IL-1ra) and IL-10, IL-6, and cartilage oligomeric matrix protein (COMP) immediately after and 1 h after the exercise session. No changes were observed in tumor necrosis factor alpha (TNF-α) and C-reactive protein (CRP). However, women with and without RA had similar changes in response to acute exercise in levels of inflammation biomarkers. In another study by Osailan A et al. [43], patients with RA underwent a treadmill exercise test. The authors investigated the heart rate recovery (HRR) and showed that HRR, which reflects parasympathetic activation, was associated with overall cardiovascular risk, arthritis-related burden, and wellbeing in RA. Bağlan Yentur S et al. [44] investigated the variation of brain-derived neurotrophic factor (BDNF) levels after acute exercise in patients with RA. The importance of BDNF is that it may promote neuronal survival, axonal guidance, and activity-dependent synaptic plasticity, and it may be associated with depression [45,46]. Moreover, BDNF plays a negative regulatory role in resolving neuroinflammation, and high inflammation reduces BDNF expression [47]. The authors found that a single bout of exercise may effectively decrease serum BDNF levels in patients with RA without, however, a possible explanation for this finding. Finally, Coelho-Oliveira AC et al. [48] investigated the effect of acute whole-body vibration exercise under the hands, on handgrip strength, range of motion, and electromyography signals of women with RA, and demonstrated that it promotes neuromuscular modifications during the handgrip of women with stable RA. On the contrary, a recent systematic literature review that investigated the acute effects of exercise on pain symptoms, clinical inflammatory markers, and inflammatory cytokines in RA concluded that post-exercise responses for pain, clinical inflammatory markers and inflammatory cytokines were not different between people with or without RA [49].

## 5. Clinical Perspectives

It is evident that more RCTs, including larger sample sizes and exercise training programs that combine both aerobic and resistance exercise, are required. There is also a need to create protocols with alternative forms of exercise for these patients who cannot tolerate aerobic exercise. Moreover, it is necessary to implement strict inclusion criteria and guidelines in order to ensure correct extraction of the results. Moderate- to high-intensity exercise, in combination with optimal medication, should be used as the main intervention in future studies in order to create personalized exercise programs with the appropriate type, intensity, and frequency of exercise training. In addition, it is important to estimate the reduction of the annual cost of hospitalizations in patients with RA of moderate–severe disease activity due to the benefits of exercise training to their daily life and the improvement of their symptoms. As a result, future research is required to formulate rehabilitation programs that improve physical condition, strength, functionality, and QoL of patients with RA.

## 6. Study Limitations

This systematic review has some limitations. Due to limited data in the literature, we could not find many clinical trials or RCTs that met our inclusion criteria. Specifically, we could not find studies including the comparison of two different modalities of exercise in RA. Furthermore, there was heterogeneity among the sample sizes of each study in terms of gender, age, and severity of the disease. Intervention was different in each study, with varying modalities of exercise training, duration of the program and the training session, intensity, and lack of follow-up after the intervention. Another limitation of our study was the fact that we did not include studies regarding the acute effects of exercise in RA. The reason was that the study design of our systematic review aimed to assess the effects of regular exercise training in RA, and not the effects of a single exercise bout.

## 7. Conclusions

This systematic review demonstrates the beneficial effects of exercise training on functional capacity and QoL in patients with RA, with an increase in peak VO_2_ and an improvement in QoL questionnaire scores after a structured exercise training program. Moreover, the reduction in pain, fatigue, and depression symptoms provided greater confidence and mobility in their daily life. Further RCTs are required in order to create individualized training protocols and find the appropriate intensity, duration, and combination of exercise modalities to achieve the maximum improvement in functional capacity and QoL.

## Figures and Tables

**Table 1:** Quality assessment of the included studies using the physiotherapy evidence database.

Criteria	Noreu et al. [21]	Rintala et al. [22]	Bilberg et al. [23]	Neuberger et al. [24]	Breedland et al. [25]	Stavropoulos-Kalinoglou et al. [26]	Azeez et al. [27]
Criterion 1: Eligibility criteria	√	√	√	√	√	√	√
Criterion 2: Random allocation		√	√	√	√	√	√
Criterion 3: Concealed allocation					√		
Criterion 4: Baseline compararability	√	√	√	√	√	√	√
Criterion 5: Blinded subjects							
Criterion 6: Blinded therapists							
Criterion 7: Blinded assesors			√	√	√		
Criterion 8: Adequate follow-up	√	√	√		√	√	
Criterion 9: Intention-to-treat analysis				√			√
Criterion 10: Between-group comparisons	√	√	√	√	√	√	√
Criterion 11: Point estimates and variability	√	√	√	√	√	√	√
Total score	4/10	5/10	6/10	6/10	7/10	5/10	5/10

Criterion 1 (eligibility criteria) is not included in the sum of the total score.

**Table 2:** Main baseline characteristics among patients with rheumatoid arthritis of each study included in the systematic review.

Studies	Groups	Females/Males (n)	Year after Diagnosis	Age (Years)	BMI (kg/m^2^)	Peak VO_2_ (mL/kg/min)	DAS-28	CPET Characteristics for All Patients (Test/Protocol)	Medication (%)
Noreau et al. [21]	I (n = 19)	12/7	8.1 ± 8.2	49.3 ± 13.0	NA	22.2 ± 7.4	NA	Maximal cycle ergometer test/Bruce protocol	Articular injections (11) Cortisone (21) NSAID (74) Remittive agents (68)
	C (n = 10)	8/2	11.0 ± 5.1	49.4 ± 11.9	NA	22.7 ± 6.5	NA		Articular injections (30) Cortisone (20) NSAID (70) Remittive agents (60)
Rintala et al. [22]	I (n = 18)	15/3	<5 (6 patients), ≥5 (12 patients)	<50 (9 patients), ≥50 (9 patients)	NA	27.9 ± 7.1	NA	Submaximal cycle ergometer test/Bruce protocol	Gold therapy (47) NSAID (100) Sulfasalazine (32) Gold therapy + sulfasalazine (6) Methotrexate (6) D-penicillamine (3) Sulfasalazine + prednisone (3) Prednisone (3) for the total sample
	C (n = 16)	14/2	<5 (6 patients), ≥5 (10 patients)	<50 (8 patients), ≥50 (8 patients)	NA	25.7 ± 5.1	NA		
Bilberg et al. [23]	I (n = 20)	NA	31 ± 15.8 *	49 (32–62)	NA	34.0 ± 10.9	4.1 ± 1.5	Submaximal cycle ergometer test/Astrand–Rhyming protocol	Analgesic (65) DMARD (90) Oral steroids (15)
	C (n = 23)	NA	35 ± 17.1 *	46 (21–65)	NA	34.2 ± 6.7	4.0 ± 1.3		Analgesic (43) DMARD (87) Oral steroids (17)
Neuberger et al. [24]	I1 (n = 68)	NA	8 (0.5–50) for total sample	55.5 (40–70) for total sample	NA	22.50 ± 9.15	NA	Submaximal cycle ergometer test/Astrand–Rhyming protocol	NSAID or DMARD as standard therapy for total sample
	I2 (n = 79)				NA	23.32 ± 7.19	NA		
	C (n = 73)				NA	21.10 ± 8.15	NA		
Breedland et al. [25]	I (n = 19)	12/7	9.7 ± 14.0	45 ± 11.9	NA	31.52 ± 10.17	2.9 ± 1.1	Submaximal cycle ergometer test/Astrand–Rhyming protocol	DMARD (21) NSAID + DMARD (68) Biological + DMARD (11)
	C (n = 15)	12/13	5.9 ± 7.2	51.8 ± 9.4	NA	25.99 ± 6.07	3.1 ± 0.9		NSAID (7) DMARD (27) NSAID + DMARD (60) No medication (7)
Stavropoulos-Kalinoglou et al. [26]	I (n = 18)	14/4	5.5 (3.0–9.7)	55.0 ± 9.8	28.7 ± 5.1	24.8 ± 7.6	3.2 ± 1.2	Maximal treadmill test/Ramp test protocol	DMARD as standard therapy for total sample
	C (n = 18)	14/4	7.0 (5.0–10.0)	52.8 ± 10.1	28.8 ± 5.3	22.4 ± 5.7	3.2 ± 1.1		
Azeez et al. [27]	I (n = 28)	24/4	2 (2–21)	58.5 (34–73)	26.1 (18–47)	24.3 (16–31.8)	2.37 (0.49–3.7)	Submaximal treadmill test/Modified Bruce protocol	DMARD (86) Biologic (61) DMARD and biologic (54) Anti-hypertensive (29) No treatment (7)
	C (n = 24)	20/4	9 (1–43)	63 (36–74)	26.3 (21–46)	25.9 (14–31.8)	2.69 (0.49–5.3)		Steroid (8) DMARD (79) Biologic (33) DMARD and biologic (29) Anti-hypertensive (38) No treatment (17)

I, intervention group; C, control group; PeakVO_2_, peak oxygen consumption; BMI, body mass index; DAS-28, disease activity score 28; CPET, cardiopulmonary exercise testing; NSAID, non-steroidal anti-inflammatory drugs; DMARD, disease-modifying antirheumatic drugs; NA, not available. * RA duration is measured in months. There were no differences between baseline parameters such as gender, age, years after diagnosis, peak VO_2_, and DAS-28 between the 2 groups in each study (*p* > 0.05).

**Table 3:** Population, intervention, comparison, outcomes, and study (PICOS) design of each study included in the systematic review.

Studies	Interventions by Group	Frequency	Session Duration	Intervention Duration	Outcomes	Main Results
Noreu et al. [21]	I: Warm-up (10 min), Exercise (15–30 min): aerobic exercise in the format of aerobic dancing, without jumps or sudden movements. 3 weeks: 50% of HRR max. 9 weeks: 70% of HRR max. Cool-down (10 min) Counseling C: usual care	Twice per wk Once per wk	35–50 min	12 wk	Peak VO_2_ QoL (AIMS, POMS)	Peak VO_2_ - ↑ in peak VO_2_ in the I group (from 22.2 ± 7.5 to 25.0 ± 7.2 mL/kg/min, *p* ≤ 0.01). In the C group, there was not a significant difference (from 22.7 ± 6.5 to 23.8 ± 5.7 mL/kg/min, *p* > 0.05). QoL AIMS - Significant ↓ in pain, mobility, household activities, depression, and anxiety within the I group (*p* < 0.05), but no difference in any of these variables within the C group (*p* > 0.05). POMS - Significant ↓ in tension, fatigue, depression, and total score, and significant ↑ in vigor within the I group (*p* < 0.01), but only in depression within the C group (*p* < 0.05).
Rintala et al. [22]	I: Water exercise program in a pool. Warm-up (12 min) Exercise: arm, trunk, and leg movements, such as rotation of the upper body, abduction and adduction of arm and legs, and flexion and extension of arms; some included the use of fins and balls (35 min). Cool-down included stretching, floating, and breathing exercises. C: usual care	2 times/wk	45–60 min Increased gradually	12 wk	Peak VO_2_	Peak VO_2_ - In the I group, increase from 27.9 + 7.1 to 29.5 ± 7.0 mL/kg/min, and in the C group, from 25.7 + 5.1 to 27.1 ± 5.8 mL/kg/min). - No statistically significant difference between groups (*p* > 0.05).
Bilberg et al. [23]	I: exercises for aerobic capacity, of moderate aerobic intensity, dynamic (eccentric and concentric), static muscle strength, and muscle endurance in the upper and lower extremities, flexibility, coordination, and relaxation. C: usual care	2 times/wk	45 min	12 wk	Peak VO_2_ QoL (SF-36, AIMS2)	Peak VO_2_ - No significant difference within the I group (from 34.0 ± 10.9 to 33.8 ± 10.0 mL/kg/min, *p* > 0.05) and the C group (from 34.2 ± 6.7 to 32.4 ± 7.3 mL/kg/min, *p* > 0.05). - No statistically significant difference between groups (*p* > 0.05). QoL SF-36 - Significant improvement in physical functioning, bodily pain, vitality, and physical component within I group (*p* < 0.01). - Significant improvement in bodily pain within C group (*p* < 0.05). - Significant improvement only in vitality in the I group compared to the C group (*p* = 0.021). AIMS2 - Significant improvement in physical and HAQ score within the I group (*p* < 0.05). - No difference within the C group (*p* > 0.05). - Significant improvement in HAQ score in the I group compared to the C group (*p* = 0.045).
Neuberger et al. [24]	I 1: Gym-based warm-up, low-impact aerobic exercise, strengthening exercises, and cool-down, 60% and 80% MHR. Weeks 1–3: 20, 10, 20, and 10 min; weeks 2–3: 15, 20, 15, and 10 min; weeks 4–6: 10, 25, 20, and 5 min; and weeks 7–12: 10, 30, 15, and 5 min. I 2: The same program, but home- based from a videotape. C: usual care	3 times/wk	60 min	12 wk	Peak VO_2_ QoL (POMS, MAF, CES-D)	Peak VO_2_ - Significant difference within the I1 group (from 22.50 to 25.09 mL/kg/min, *p* < 0.05) and I2 group (from 23.32 to 24.58 mL/kg/min, *p* < 0.05), but not within the control group (*p* > 0.05). - No statistically significant difference between the exercise and control groups (*p* > 0.05). QoL - Overall symptoms (latent variable for pain, fatigue, and depression) decreased significantly (*p* < 0.04) within the I1 and I2 groups compared to the C group, as well as between the I and C groups.
Breedland et al. [25]	I: Muscle exercise circuit at 40–60% of 1 RM for 3 sets × 20 reps with increased load 5%/wk. Bicycle training, 10–20 min, at 60% HRmax. Other activities included badminton, table tennis, bowling, uni-hockey, circuit training, and aqua jogging. C: usual care	2 times/wk	90 min	8 wk	Peak VO_2_ DAS28 QoL (Dutch-AIMS2, ASES)	Peak VO_2_ - ↑ in the I group (from 31.52 ± 10.17 to 35.34 ± 11.33 mL/kg/min, *p* < 0.01), while no significant change in the C group (from 25.99 ± 6.07 to 25.55 ± 6.36 mL/kg/min, *p* = 0.24). - There was a statistically significant increase in the I group compared to the C group (+12.1% versus −1.7%, *p* = 0.002). DAS28 No significant differences in DAS28 scores between groups. QoL Dutch-AIMS2 - Significant improvement in physical health within the I group (*p* = 0.05); no significant changes were found within the C group (*p* > 0.05). - No statistically significant differences between groups (*p* > 0.05). ASES - No statistically significant differences within and between groups (*p* > 0.05).
Stavropoulos- Kalinoglou et al. [26]	I: First 3 months aerobic exercise. Three circuits of 3–4 exercises (walk on treadmill, cycle, row, or hand ergometer) at 70% VO_2_max for 3–4 min, with 1 min resting interval. After 3 months, resistance training was added to the schedule above. Three sets of 4 resistance exercises (leg press, chest press, shoulder press, pull-ups) for 12–15 reps. C: usual care	3 times/wk	50–60 min	6 months	Peak VO_2_ DAS28 QoL (HAQ) At 3 and at 6 months	Peak VO_2_ - Peak VO_2_ significantly improved within the I group from 24.8 ± 7.6 mL/min/kg by >10% and 17% within 3 and 6 months (*p* < 0.001), while there was not an improvement within the C group (*p* > 0.05). - There was a significant increase in the I group compared to the C group (3 months: *p* = 0.023; 6 months: *p* = 0.002). DAS28 - ↓ DAS28 within the I group (from 3.5 ± 1.2 to 2.9 ± 0.8 at 3 months and 2.7 ± 0.7 at 6 months; *p* < 0.05), but no significant reduction within the C group (from 3.1 ± 1.2 to 3.1 ± 0.6 at 3 months and 3.2 ± 0.9 at 6 months; *p* > 0.05). - Statistically significant improvement in the I group compared to the controls (*p* = 0.008). QoL (HAQ) - Significant improvement in the I group (from 1.4 ± 0.8 to 1.0 ± 0.6 at 3 months and 0.9 ± 0.6 at 6 months; *p* < 0.001), but not in the C group (from 1.3 ± 0.7 to 1.6 ± 0.5 at 3 months and 1.5 ± 0.6 at 6 months; *p* > 0.05). - Significant improvement in the I group compared to the C group (*p* = 0.003).
Azeez et al. [27]	I: Personalized exercise program. Cardiovascular exercises, such as walking, cycling, or swimming, depended on the patient’s preferences. Strength exercises for the upper body (biceps curls, triceps extensions, and shoulder press). Exercises for the lower body (leg squat). Resistance bands and balls were used for grip strength. C: usual care	NA	NA	3 months	Peak VO_2_ DAS28 QoL (HAQ, GFI)	Peak VO_2_ - Significant increase within the I group (from 23.2 (16–88) to 27.6 (14–75) mL/kg/min, *p* = 0.002), but no difference in the C group (from 26.1 (14–83) to 27.6 (18–65) mL/kg/min, *p* = 0.313). DAS28 No difference in DAS28 within and between groups (*p* > 0.05). QoL HAQ - Significant improvement in HAQ within the I group (from 0.5 (0.0–2.4) to 0.25 (0.0–2.5), *p* = 0.05) and within the C group (from 1.1 (0–3.0) to 0.8 (0.0–2.9), *p* = 0.026). - No difference between groups (*p* > 0.05). GFI (global fatigue index) - Significant improvement within the I group (from 13.2 (6.4–34.1) to 10.9 (6.5–37.5), *p* = 0.047), but no difference within the C group (*p* = 0.96). - Significant improvement in the I group compared to the C group (*p* = 0.047).

HRR max, maximal heart rate reserve; MHR, maximum heart rate; QoL, quality of life; wk, weeks; min, minutes; 1 RM, one-repetition maximum; AIMS, Abnormal Involuntary Movement Scale (questionnaire); AIMS2, Arthritis Impact Measurement Scales 2 (questionnaire); Dutch-AIMS2, Arthritis Impact Measurement Scales Dutch version (questionnaire); POMS, Profile of Mood States (questionnaire); SF-36, 36-Item Short-Form Survey (questionnaire); HAQ, The Health Assessment Questionnaire; GFI, Global Fatigue Index (questionnaire). ↑ means increase and ↓ means decrease.

## Data Availability

No new data were created.
